# Allopurinol and the risk of stroke in older adults receiving medicare

**DOI:** 10.1186/s12883-016-0692-2

**Published:** 2016-09-07

**Authors:** Jasvinder A. Singh, Shaohua Yu

**Affiliations:** 1 Medicine Service, Birmingham VA Medical Center, Birmingham, AL USA; 2 Department of Medicine at School of Medicine, University of Alabama at Birmingham (UAB), Birmingham, AL USA; 3 Division of Epidemiology at School of Public Health, University of Alabama at Birmingham (UAB), Birmingham, AL USA; 4 Department of Orthopedic Surgery, Mayo Clinic College of Medicine, Rochester, MN USA; 5 University of Alabama, Faculty Office Tower 805B, 510 20th Street S, Birmingham, AL 35294 USA

**Keywords:** Allopurinol, Stroke, Elderly, Medicare, Ischemic stroke, Urate-lowering therapy, Hyperuricemia, Age, Race, Gender

## Abstract

**Background:**

Previous studies of allopurinol and stroke risk have provided contradictory findings, ranging from a protective effect to an increased risk. Our objective was to assess whether allopurinol use is associated with the risk of stroke in the elderly.

**Methods:**

We used the 5 % random sample of Medicare beneficiaries from 2006–2012 to study the association of new allopurinol initiation and incident stroke. We used multivariable-adjusted Cox regression models adjusted for age, gender, race, Charlson index, and cardio-protective medications (beta-blockers, ACE inhibitors, diuretics, statins) to calculate hazards ratio (HR) with 95 % confidence intervals (CI). Sensitivity analyses adjusted for coronary artery disease (CAD) risk factors including hypertension, hyperlipidemia, diabetes, and smoking instead of Charlson index.

**Results:**

Among 28,488 eligible episodes of incident allopurinol, 2,177 ended in incident stroke (7.6 % episodes). In multivariable-adjusted analyses, allopurinol use was associated with 9 % lower hazard ratio for stoke, 0.91 (95 % CI, 0.83 to 0.99). Compared to no allopurinol use, allopurinol use durations of 181 days to 2 years, 0.88 (95 % CI, 0.78 to 0.99) and >2 years, 0.79 (95 % CI, 0.65 to 0.96) were significantly associated with lower multivariable-adjusted hazard of stroke. Sensitivity analyses adjusted for CAD risk factors confirmed these findings. In subgroup analyses, significant associations were noted between allopurinol use and the risk of ischemic stroke, 0.89 (95 % CI, 0.81 to 0.98); associations were not significant for hemorrhagic stroke, 1.01 (95 % CI, 0.79 to 1.29).

**Conclusions:**

Allopurinol use is associated with lower risk of stroke overall, more specifically ischemic stroke. This association is evident after 6-months of allopurinol use, and the hazard reduction increases with longer duration of use. Future studies need to examine underlying mechanisms.

**Electronic supplementary material:**

The online version of this article (doi:10.1186/s12883-016-0692-2) contains supplementary material, which is available to authorized users.

## Background

Stroke is the second leading cause of cardiovascular mortality worldwide and among the top five causes of death in the U.S. [1, 2]. In the U.S., 6.4 million people, 2.7 % of the adult population, suffered from stroke, according to the 2012 National Health Interview Survey [3]. Stroke is associated with significant disability, and a negative impact on function and quality of life [4–7]. The treatment for stroke has rapidly evolved over time, and outcomes continue to improve [8]. While some of the disease risk factors for stroke are well known, there is a relative lack of studies assessing whether certain commonly used medications can modify the risk of stroke.

Allopurinol is a urate-lowering therapy (ULT) that is commonly used for the treatment of hyperuricemia [9, 10]. In addition to its urate-lowering effect related to of the inhibition of xanthine oxidase by its active metabolite, oxypurinol, recent studies have suggested other mechanisms of actions of allopurinol, some dependent and some independent of xanthine oxidase inhibition [11–18]. Many of these effects including improvement of endothelial function and reduction of oxidative stress [14, 19–23], reduction of glycosylated hemoglobin [14], and attenuation of intercellular adhesion molecule-1 levels [24], may potentially lower the risk of stroke. To our knowledge, it is not known whether allopurinol use is associated with a lower risk of stroke.

Previous studies of allopurinol examined a composite cardiovascular outcome (that included stroke) and provided contradictory findings, ranging from a protective effect [25, 26] to an increased risk [27, 28]. These contradictory findings were related at least partially to differences in patient populations (renal failure, hypertensive nephropathy, heart failure vs. gout), study design (randomized vs. observational) and covariates/confounders adjusted in the analyses. In a 7-year follow-up of a 2-year randomized trial in patients with chronic kidney disease, adjusted for race, sex and renal function, allopurinol use was associated with lower risk of 0.43 (95 % CI, 0.21 to 0.88) of cardiovascular events (including CAD, cerebrovascular disease, heart failure and peripheral vascular disease) [25]. In an observational study of 187 patients with hypertensive nephropathy, allopurinol use was associated with an adjusted hazard of 0.34 (*p* = 0.04) for cardiovascular disease (CAD, heart failure and stroke) [26]. In a study of heart failure patients using Scottish database, Wei et al. reported that with a minimum follow-up of 5 years, compared with non-users, allopurinol users had a no significant difference in cardiovascular event (nonfatal myocardial infarction (MI), nonfatal stroke and cardiovascular mortality), 0.88 (95 % CI, 0.73 to 1.05) [27]. Kok et al. [28] studied >4,000 patients with newly diagnosed gout and no pre-existing severe form of CVD using a prevalent user design and reported that allopurinol use was associated with an increased adjusted hazard of 1.25 for cardiovascular event (hospitalization for MI, stroke, hypertension etc.).

None of the previous studies examined the association of allopurinol with stroke as an outcome in the general population. Therefore, findings can only be generalized to patient sub-populations with hypertensive nephropathy, renal failure, gout or heart failure, and then only to a cardiovascular composite outcome, that includes peripheral vascular disease, heart failure, MI, angina etc. in addition to stroke. Thus, well-designed studies examining the effect of allopurinol use on the risk of stroke are needed, especially for adults 65 years and older. This is important since 2/3^rd^ of patients with stroke in the U.S. are adults 65 years and older [29]. Therefore, our objective was to assess whether incident allopurinol use was independently associated with a reduction in the risk of incident stroke in the elderly, using a population-based approach. We hypothesized that allopurinol use and its duration of use will each be independently associated with a reduction in the risk of incident stroke in adults 65 years and older. In exploratory analyses, we examined whether stroke risk reduction with allopurinol varied by age, gender and race.

## Methods

### Study cohort and population of interest

We conducted a retrospective cohort study using the 5 % random sample of Medicare beneficiary claims from 2006 to 2012. These data were obtained from the Centers for Medicare and Medicaid Services (CMS) Chronic Condition Data Warehouse and included beneficiaries’ demographic information and insurance claims (inpatient, outpatient, skilled nursing facility, non-institutional provider, home health, hospice, durable medical equipment and prescription drugs).

Study eligibility criteria were: (1) Medicare beneficiaries who were 65 years of age or older and lived in the U.S.; (2) continuous enrollment in traditional Medicare fee-for-service and pharmacy coverage (Parts A, B and D) and non-enrollment in Medicare Advantage Plan; and (3) new treatment with allopurinol (defined below). The Institutional Review Board at the University of Alabama at Birmingham approved the study, and waived the need for patient informed consent for this database study. All investigations were conducted in conformity with ethical principles of research.

### Exposure definition and covariates

Incident allopurinol use, i.e. new allopurinol use, was defined as a new filled allopurinol prescription, with no allopurinol prescription filled during a look-back baseline period of 365 days. Each day of observation within each episode was labeled as allopurinol exposed or non-exposed based upon the days’ supply for allopurinol prescription in pharmacy records after the beginning of allopurinol treatment episode. We allowed up to 30 days stock carry over. Patients were considered exposed for 30 days after the end of days’ supply to capture events attributable to allopurinol, after which a new continuous allopurinol exposure period started. Allopurinol use duration was categorized as none, 1 to 180 days, 181 days to 2 years, and longer than 2 years, based on clinical relevance and current use patterns of allopurinol [9, 10]. A patient could contribute multiple allopurinol treatment episodes during different time periods.

We obtained the following covariates in the baseline period for each allopurinol treatment episode from the Medicare denominator file: age, gender, race/ethnicity, residence (Northeast, South, Midwest, West) and comorbidity scores, derived using Charlson-Romano comorbidity index score, a validated score for comorbidity assessment consisting of common medical comorbidities [30], adapted for claims data by Romano et al.. Comorbidities included in Charlson-Romano comorbidity index were: myocardial infarction, congestive heart failure, cerebrovascular disease, dementia, chronic pulmonary disease, connective tissue disease, peptic ulcer disease, mild liver disease, diabetes without complications, diabetes with complications, paraplegia and hemiplegia, renal disease, cancer, moderate to severe liver disease, metastatic cancer and AIDS/HIV [31]. Charlson-Romano index score is a valid comorbidity measure [32, 33].

### Study outcome

Incident stroke was our study outcome of interest, defined as the first occurence of stroke during the study period after the initiation of a new allopurinol prescription. Incident stroke was identified by the presence of International Classification of Diseases, ninth revision, common modification (ICD-9-CM) codes for stroke (430.xx, 431.xx, 433.x1, 436.xx, 434.xx, except 434.x0); subgroup analyses were for hemorrhagic stroke (430.xx, 431.xx) and ischemic stroke (rest of the codes). This approach using ICD-9-CM codes is accurate for identification of cases and risk factors, and has high positive predictive value, usually exceeding >90 % [34–36]. The earliest allopurinol treatment initiation date during the study period marked the beginning of the follow-up for each treatment episode that ended on the earliest of first date of stroke, losing full Medicare coverage, the date of death, or the end of the study (12/31/12).

### Statistical analyses

We calculated descriptive statistics by the occurrence of stroke among allopurinol users and by allopurinol exposure among episodes with stroke. The main analysis focused on stroke (including all subtypes). We performed Cox proportional hazard regression models to assess the association between allopurinol exposure or duration of allopurinol use and incident stroke. Multivariable analysis were adjusted for age, gender, race and Charlson-Romano comorbidity score. We accounted for correlated episodes using the Huber-White “Sandwich” variance estimator and calculated robust standard errors for all estimates, since patients could possibly contribute more than one episode of new allopurinol use. We performed sensitivity analyses using Cox proportional hazard regression models to assess these associations by replacing Charlson-Romano index score with selected comorbidities, including risk factors for coronary artery disease (CAD) in one model, and replacing with  peripheral vascular disease (PVD) and CAD in another separate model. We calculated hazard ratio (HR) and 95 % confidence intervals (CI). We also performed subgroup analyses by assessing the relationship of allopurinol use and allopurinol use duration to each type of stroke, ischemic vs. hemorrhagic stroke.

## Results

### Study population characteristics

**Fig. 1:**
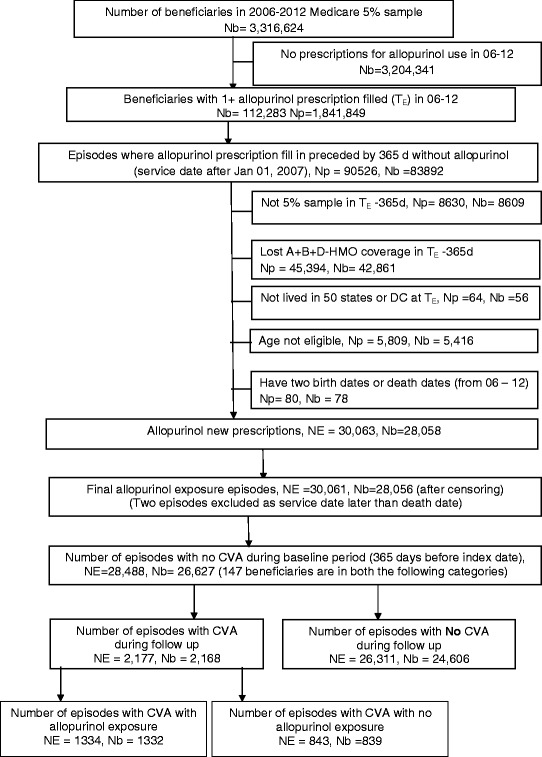
Flow-chart of study cohort of incident allopurinol users from 2006–2012 for baseline of 365 days. Legend: NE, number of episodes; Nb, number of beneficiaries; Np, number of prescriptions

There were a total of 28,488 episodes of incident allopurinol use (Table 1). Of these, 2,177 allopurinol use episodes were associated with incident stroke (7.6 % episodes; baseline period of 365 days without any stroke), while the majority did not (Fig. 1). Compared to incident allopurinol use without stroke, those with episodes of incident stroke on allopurinol were older, more likely to be women or white, and had higher Charlson index score (4.27 vs. 3.56); some differences were noted by region of residence (Table 1). Mean (median) length to first stroke event was 437 (median, 299) days and mean (median) follow-up time was 704 (median, 579) days.

**Table 1:** Demographic and clinical characteristics of episodes of incident^a^ allopurinol use

	All	Incident stroke during follow up		*P*-value
		No	Yes	
Total, N (episodes)	28,488	26,311	2,177	
Age	76.5 (7.4)	76.4 (7.4)	78.1 (7.3)	<0.0001
Gender, N (%)				<0.0001
Male	14,163 (49.7)	13,185 (50.1)	978 (44.9)	
Female	14,325 (50.3)	13,126 (49.9)	1,199 (55.1)	
Race/Ethnicity, N (%)				0.0004
White	22,627 (79.4)	20,970 (79.7)	1,657 (76.1)	
Black	3,371 (11.8)	3,049 (11.6)	322 (14.8)	
Hispanic	589 (2.1)	538 (2.0)	51 (2.3)	
Asian	1,236 (4.3)	1,134 (4.3)	102 (4.7)	
Native American	92 (0.3)	83 (0.3)	9 (0.4)	
Other/unknown	573 (2.0)	537 (2.0)	36 (1.7)	
Region, N (%)				0.013
Northeast	4,586 (16.6)	4,224 (16.6)	362 (17.3)	
Midwest	6,400 (23.2)	5,958 (23.3)	442 (21.1)	
South	11,405 (41.3)	10,486 (41.1)	919 (44.0)	
West	5,227 (18.9)	4,859 (19.0)	368 (17.6)	
Charlson- Romano comorbidity Index Score	3.61 (3.22)	3.56 (3.20)	4.27 (3.39)	<0.0001

^a^No allopurinol use in the baseline period of 365 days

Table 2 shows the crude incidence rate of stroke by allopurinol exposure. Crude incidence rates of stroke with and without allopurinol exposure were similar. Durations of allopurinol use longer than 6-month were associated with lower crude incidence rate of stroke compared to no allopurinol use (Table 2).

**Table 2:** Crude incidence rate of CVA with allopurinol exposure and allopurinol use duration

	Person-days of follow up	Stroke cases	Crude Stroke Incidence Rate per 1,000,000 person-days
Allopurinol Exposure^a^			
Yes	12,900,000	1,334	108
No	7,902,385	843	106
Allopurinol use duration			
None	7,902,385	843	106
1 to 180 days	5,676,393	728	128
181 days to 2 year	4,876,688	476	98
> 2 years	1,721,507	130	76

^a^Allopurinol exposure was defined as up to 30 days after last day of allopurinol prescription fill or refill to capture the biologic effect of allopurinol use

### Allopurinol and the risk of incident stroke

Compared to non-use, age and Charlson comorbidity index were higher with allopurinol use for episodes with incident stroke (Table 3). In multivariable-adjusted analyses, allopurinol use was associated with 9 % lower hazard ratio for stoke, 0.91 (95 % CI, 0.83 to 0.99). Age 75- < 85 and ≥85, black race, higher Charlson-Romano index score and the use of beta-blockers were associated with higher hazards of incident stroke (Table 4). Specific hazards ratios by age, gender and race are shown in Additional file 1: Table S1.

**Table 3:** Demographic and comorbidity characteristics of episodes that ended in stroke by allopurinol use

	Episodes with stroke		*P*-value
	Not on Allopurinol (NE = 843)	On Allopurinol (NE = 1,334)	
Age	77.5 (7.1)	78.5 (7.4)	0.003
Gender			0.95
Male	378 (44.8)	600 (45.0)	
Female	465 (55.2)	734 (55.0)	
Race			0.54
White	632 (75.0)	1,025 (76.8)	
Black	128 (15.2)	194 (14.5)	
Others	83 (9.9)	115 (8.6)	
Charlson-Romano Index Score	3.99 (3.27)	4.44 (3.44)	0.002
Specific Comorbidities			
Diabetes	382 (45.3)	641 (48.1)	0.21
Hypertension	724 (85.9)	1,174 (88.0)	0.15
Cardiovascular Disease	128 (15.2)	232 (17.4)	0.18
Peripheral Vascular Disease	187 (22.2)	302 (22.6)	0.80
Hyperlipidemia	591 (70.1)	970 (72.7)	0.19

*NE* number of episodes

**Table 4:** Association of risk factors with incident stroke^a^ in patients who received allopurinol

	Univariate		Multivariable-adjusted (model 1)*		Multivariable-adjusted (model 2)**	
	HR (95 % CI)	*P*-value	HR (95 % CI)	*P*-value	HR (95 % CI)	*P*-value
Age						
65- <75	Ref		Ref		Ref	
75- <85	1.63 (1.48, 1.79)	<0.0001	1.54 (1.40, 1.70)	<0.0001	1.54 (1.40, 1.70)	<0.0001
≥ 85	2.09 (1.86, 2.35)	<0.0001	1.91 (1.69, 2.15)	<0.0001	1.91 (1.69, 2.16)	<0.0001
Gender						
Male	Ref		Ref		Ref	
Female	1.21 (1.12, 1.32)	<0.0001	1.09 (1.00, 1.19)	0.05	1.09 (1.00, 1.19)	0.05
Race						
White	Ref		Ref		Ref	
Black	1.40 (1.24, 1.57)	<0.0001	1.37 (1.21, 1.54)	<0.0001	1.36 (1.21, 1.53)	<0.0001
Other	1.09 (0.94, 1.26)	0.27	1.09 (0.94, 1.27)	0.23	1.09 (0.94, 1.26)	0.26
Charlson- Romano score	1.11 (1.10, 1.12)	<0.0001	1.10 (1.09, 1.11)	<0.0001	1.10 (1.09, 1.11)	<0.0001
Cardiovascular drug use (Ref, no use)						
Statins	0.93 (0.75, 1.15)	0.49	0.91 (0.73, 1.13)	0.39	0.91 (0.73, 1.13)	0.38
Beta blockers	1.38 (1.15, 1.67)	0.0007	1.40 (1.15, 1.70)	0.0008	1.40 (1.15, 1.70)	0.0008
Diuretics	1.05 (0.86, 1.27)	0.63	0.97 (0.79, 1.18)	0.74	0.96 (0.79, 1.18)	0.72
ACE inhibitor	0.92 (0.73, 1.18)	0.52	0.97 (0.76, 1.24)	0.78	0.97 (0.76, 1.23)	0.78
Allopurinol use (Ref, none)	0.92 (0.84, 1.01)	0.07	0.91 (0.83, 0.99)	0.04	-	-
Allopurinol use duration						
None	Ref				Ref	
1 - 180 days	1.02 (0.89, 1.15)	0.81			1.00 (0.88, 1.14)	0.97
181 days - 2 years	0.90 (0.80, 1.01)	0.07			0.88 (0.78, 0.99)	0.03
> 2 years	0.78 (0.64, 0.96)	0.02			0.79 (0.65, 0.96)	0.02

* Model 1 = Allopurinol use + age + race + gender + Charlson score + beta blockers + diuretics + ACE inhibitors + Statins

** Model 2 = Allopurinol use duration + age + race + gender + Charlson score + beta blockers + diuretics + ACE inhibitors + Statins

^a^No stroke within the baseline period of 365 days before the index date of allopurinol episode

We found that compared to no allopurinol use, allopurinol use durations of longer than 6-months were each associated with lower multivariable-adjusted hazard ratios for stroke: 181 days to 2 years, 0.88 (95 % CI, 0.78 to 0.99) and >2 years, 0.79 (95 % CI, 0.65 to 0.96) (Table 4).

Sensitivity analyses were performed adjusting for diabetes, hypertension, hyperlipidemia and tobacco use disorder as CAD risk factors, peripheral vascular disease and CAD confirmed the findings for both allopurinol use and the duration of allopurinol use (Additional file 2: Table S2).

### Subgroup analyses for allopurinol use duration and by the type of stroke

**Fig. 2:**
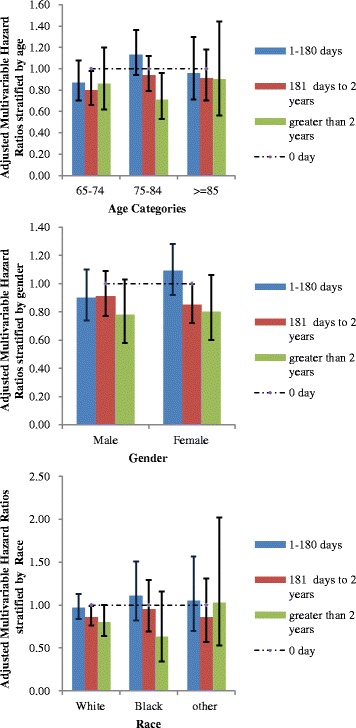
Multivariable-adjusted* Hazard ratios of duration of allopurinol use with incident stroke by age (2a), gender (2b) and race (2c). Legend: *For the multivariable-adjusted subgroup analyses by age, gender and race, the main model was adjusted for all factors (age, gender, race and Charlson-Romano comrobidity score) except the factor of interest, respectively, which was used to perform stratified analysis (age, gender, race). We found that several subgroups had statistically significantly reduced hazard with allopurinol use, namely: Age group 65–75 years, 181 days to 2 years; Age group 75–84 years, >2 years; female gender, 181 days to 2 years; White race, 181 days to 2 years and >2 years. As expected, most of the subgroups had very few events, and therefore most subgroup analyses did not have power to detect significant differences within each subgroup

In multivariable-adjusted subgroup analyses, allopurinol use durations of 181 days to 2 years and >2 years were associated with a reduction of hazard of stroke (Table 4), most evident for the age group 75–84, female gender and patients who were white (Fig. 2).

In multivariable-adjusted subgroup analyses, we found no significant association of allopurinol use or duration of allopurinol use with the risk of hemorrhagic stroke (Additional file 3: Table S3). On the other hand, significant associations were noted between allopurinol use and the risk of ischemic stroke, similar in magnitude to overall incident stroke analyses (Additional file 3: Table S3).

## Discussion

We used an incident/new user design (new users of allopurinol indicated by a new allopurinol filled prescriptions) and a population-based approach to examine the effect of allopurinol use on the risk of stroke in the U.S. elderly. We controlled for patient demographics, comorbidity and use of cardio-protective medications to understand whether the association of allopurinol was independent of other CAD risk factors and treatments. This new knowledge is important, since the majority of strokes occur in adult Americans 65 years and older [29]. Subgroup analyses examined whether association of allopurinol was evident for separate analyses for ischemic and hemorrhagic stroke. Several study findings merit further discussion.

Our finding of independent association of incident allopurinol use with a reduction of hazard of incident stroke in the older adults compared to no allopurinol use, is novel. We are unaware of other studies that have examined this specific question. Most previous studies in this area of research had several limitations in that: (1) all studied a composite cardiovascular outcome that included heart failure, angina, MI etc. in addition to stroke [25–28]; (2) most used prevalent user design [26, 28]; and (3) all studies were in patient subpopulations with limited generalizability [25–28]. Composite outcome assessment did not allow the interpretation regarding the risk of stroke, due to inclusion of related outcomes, which might have different mechanisms than stroke, i.e., heart failure, angina etc. It is therefore not surprising that findings ranged from a reduced risk of with allopurinol [25, 26] to an increased risk of composite cardiovascular outcome that included stroke [27, 28].

The stroke risk reduction associated with allopurinol use noted in our study and others may be related to several potential mechanisms. Allopurinol use was associated with attenuation of intercellular adhesion molecule-1 levels in patients after a recent stroke [24]. Allopurinol use improved endothelial function and reduction of oxidative stress [14, 19–23] and reduced glycosylated hemoglobin [14]. Allopurinol reduced serum urate, and an elevated serum urate was associated with higher levels of chronic inflammatory markers in cardiac disease [37].

Regardless of the mechanism of action, the significant reduction of stroke risk associated with allopurinol has practical implications. The hazards of stroke were reduced by 9 % for allopurinol use vs. non-use and reduced by 12–21 % with longer allopurinol use durations. Thus, the magnitudes of hazard reduction were moderate, not large. Allopurinol is commonly used for the treatment of gout and reduced morbidity related to joint inflammation and pain, including disability, functional limitation and joint destruction. The potential added benefit of stroke reduction with allopurinol should be shared with patients with gout at the time of allopurinol treatment initiation. This discussion should impact the overall risk/benefit ratio of allopurinol, as well as serve as a motivating factor for improving allopurinol adherence and persistence, which is low for allopurinol in the first year after initiation [38]. More evidence is needed before an informed benefit/risk analysis of allopurinol use can be shared with patients with asymptomatic hyperuricemia without gout, where allopurinol use is currently not recommended [39].

Maximum hazard reduction in incident stroke with allopurinol use occurred in the >6 to 24 months and >2 years after new allopurinol use, with 12 % and 21 % hazard reduction, respectively. We noted an incremental stroke risk reduction associated with longer duration of allopurinol use. This important finding indicates that allopurinol use is needed for >6 months to experience this potential benefit.

In exploratory multivariable-adjusted subgroup analyses, allopurinol use durations of 181 days to 2 years and >2 years were associated with a reduction of hazard of stroke, most evident for the age group 75–84, female gender and people who were white. Our study identified the sub- groups of patients likely to benefit the most with longer durations of allopurinol use. These findings need to be confirmed in future studies.

Our exploratory analyses added further clarity to this protective effect of allopurinol. Risk reduction associated with allopurinol use was noted for ischemic stroke, but not hemorrhagic stroke. These two types of stroke differ quite a bit in pathophysiology and outcomes [40]. It was reassuring that the protection for overall stroke risk is primarily due to protection against ischemic stroke, which is consistent with allopurinol’s effect on oxidative stress, endothelial function and chronic inflammation [14, 19–23, 37].

Our study has several strengths and limitations. The diagnoses of CAD risk factors and Charlson-Romano index were based on the presence of ICD-9-CM codes, which may have led to misclassification bias. However, the codes for outcomes and covariates have reasonable accuracy [34–36], and the use of ICD-9-CM codes for Charlson-Romano index is a standard valid approach that is used commonly in analyses of large claims databases, such as Medicare. The observational study design does not allow us to eliminate confounder bias, due to unmeasured confounding; however, we included important demographic, clinical, risk factors and cardiac medications to account for important potential confounders. Serum urate levels were available for a small proportion of patients and therefore we could not examine whether the associations we noted were mediated through the reduction of serum urate or not. The database also did not allow us to include other variables such body mass index, diet and exercise, which might be potential risk factors for stroke.

The contribution of healthy adherer effect can not be ruled out, since patients adherent to/using allopurinol may have other healthy behaviors, such as high adherence to preventive care, other medications, healthier lifestyle etc., which may all reduce the risk of stroke etc. A filled prescription is a surrogate for, but can not be equated to the actual use of allopurinol. Since study’s eligibility criteria included that patients to have continued coverage, these findings are likely not applicable to elderly who loose Medicare coverage. Whether these findings can be generalized to populations other than adults 65 years and older is not known, and further study is needed in this area.

Study strengths include a large sample size, the use of a sample that allows generalizable findings to Medicare enrollees, inclusion of MI/stroke risk factors in analyses to avoid confounding bias, and the robustness of estimates in sensitivity analyses. Implementation of new user design (i.e., examining only new users of allopurinol) in our study avoids over-adjustment for factors on the causal pathway, unlike the prevalent user design, used in previous studies.

## Conclusions

In conclusion, we found that allopurinol use was independently associated with a lower risk of incident stroke in older Americans. Allopurinol use for 6 months to 2 years and >2 years were associated with a lower risk of incident stroke. The stroke risk reduction associated with allopurinol was seen in the subgroup analysis for ischemic stroke, but not hemorrhagic stroke. Future studies need to explore the factors that mediate this potentially protective effect, and assess whether these factors are dependent or independent of serum urate reductions and anti-oxidant effects seen with allopurinol.

## Additional files

Additional file 1: Table S1. Multivariable-adjusted* Hazard ratios of allopurinol use with incident stroke by age (a), gender (b) and race (c). (DOCX 79 kb) Additional file 2: Table S2. Sensitivity analysis: Association of various risk factors with incident stroke* in patients who received allopurinol with cardiovascular disease and associated risk factors replacing Charlson score. (DOCX 161 kb) Additional file 3: Table S3. Subgroup analyses by the type of stroke. (DOCX 89 kb)

## Abbreviations

ACE inhibitor: Angiotensin converting enzyme inhibitor
CAD: Coronary artery disease
ED: Emergency department
ICD-9-CM: International classification of diseases, ninth revision, common modification codes
COPD: Chronic obstructive pulmonary disease
NEDS: National ED sample
ULT: Urate-lowering therapy
