# Supplementary material for: Allopurinol and the risk of stroke in older adults receiving medicare
Source: BMC Neurol. 2016 Sep 7;16(1):164. doi: 10.1186/s12883-016-0692-2 (PMC5015204; doi:10.1186/s12883-016-0692-2)
Supplement: Additional file 1: Table S1. — Multivariable-adjusted* Hazard ratios of allopurinol use with incident stroke by age (a), gender (b) and race (c). (DOCX 79 kb) [file 12883_2016_692_MOESM1_ESM.docx]

**Supplementary table 1**. **Multivariable-adjusted* Hazard ratios of allopurinol use with incident stroke by age (a), gender (b) and race (c).**

**1A. Age**

**1B. Gender**

**1C. Race**
