# Supplementary material for: Allopurinol and the risk of stroke in older adults receiving medicare
Source: BMC Neurol. 2016 Sep 7;16(1):164. doi: 10.1186/s12883-016-0692-2 (PMC5015204; doi:10.1186/s12883-016-0692-2)
Supplement: Additional file 2: Table S2. — Sensitivity analysis: Association of various risk factors with incident stroke* in patients who received allopurinol with cardiovascular disease and associated risk factors replacing Charlson score. (DOCX 161 kb) [file 12883_2016_692_MOESM2_ESM.docx]

**Additional file 2**. Sensitivity analysis: Association of various risk factors with incident stroke* in patients who received allopurinol with cardiovascular disease and associated risk factors replacing Charlson score

|  | Univariate | | Multivariable-adjusted (model1) | | Multivariable-adjusted (model2) | |
| --- | --- | --- | --- | --- | --- | --- |
|  | HR (95% CI) | P-value | HR (95% CI) | P-value | HR (95% CI) | P-value |
| **Age** |  |  |  |  |  |  |
| 65- <75 | Ref |  | Ref |  | Ref |  |
| 75- <85 | **1.63 (1.48, 1.79)** | **<0.0001** | **1.57 (1.43, 1.73)** | **<0.0001** | **1.57 (1.43, 1.73)** | **<0.0001** |
| ≥85 | **2.09 (1.86, 2.35)** | **<0.0001** | **1.98 (1.75, 2.24)** | **<0.0001** | **1.98 (1.75, 2.24)** | **<0.0001** |
| **Gender** |  |  |  |  |  |  |
| Male | Ref |  | Ref |  | Ref |  |
| Female | **1.21 (1.12, 1.32)** | **<0.0001** | 1.08 (0.99, 1.18) | 0.08 | 1.08 (0.99, 1.18) | 0.08 |
| **Race** |  |  |  |  |  |  |
| White | Ref |  | Ref |  | Ref |  |
| Black | **1.40 (1.24, 1.57)** | **<0.0001** | **1.40 (1.24, 1.58)** | **<0.0001** | **1.39 (1.24, 1.57)** | **<0.0001** |
| Other | 1.09 (0.94, 1.26) | 0.27 | 1.07 (0.92, 1.24) | 0.38 | 1.06 (0.92, 1.23) | 0.42 |
| **Comorbidities** |  |  |  |  |  |  |
| Diabetes | **1.31 (1.21, 1.43)** | **<0.0001** | **1.30 (1.19, 1.41)** | **<0.0001** | **1.30 (1.19, 1.41)** | **<0.0001** |
| Hypertension | **1.29 (1.13, 1.46)** | **<0.0001** | 1.09 (0.96, 1.25) | 0.19 | 1.09 (0.96, 1.25) | 0.18 |
| PVD | **1.60 (1.44, 1.77)** | **<0.0001** | **1.30 (1.17, 1.45)** | **<0.0001** | **1.30 (1.17, 1.45)** | **<0.0001** |
| CVD | **2.39 (2.13, 2.67)** | **<0.0001** | **2.14 (1.90, 2.42)** | **<0.0001** | **2.14 (1.90, 2.42)** | **<0.0001** |
| Hyperlipidemia or statin use | 0.97 (0.88, 1.06) | 0.48 | **0.88 (0.80, 0.97)** | **0.01** | **0.88 (0.80, 0.97)** | **0.01** |
| **Tobacco Disorder** | 1.10 (0.84, 1.44) | 0.48 | 1.14 (0.87, 1.49) | 0.35 | 1.14 (0.87, 1.49) | 0.35 |
| **Other Drugs (Ref,** no use**)** |  |  |  |  |  |  |
| Beta blockers | **1.38 (1.15, 1.67)** | **0.0007** | **1.39 (1.15, 1.68)** | **0.0008** | **1.38 (1.14, 1.68)** | **0.0009** |
| Diuretics | 1.05 (0.86, 1.27) | 0.63 | 0.97 (0.79, 1.19) | 0.76 | 0.97 (0.79, 1.18) | 0.74 |
| ACE inhibitor | 0.92 (0.73, 1.18) | 0.52 | 0.92 (0.72, 1.18) | 0.51 | 0.92 (0.72, 1.18) | 0.51 |
| **Allopurinol use (Ref,** none**)** | **0.92 (0.84, 1.01)** | **0.07** | **0.91 (0.83, 0.99)** | **0.04** | - | - |
| **Allopurinol use duration** |  |  |  |  |  |  |
| 0 days | Ref |  |  |  | Ref |  |
| 1 - 180 days | 1.02 (0.89, 1.15) | 0.81 |  |  | 1.00 (0.88, 1.13) | 0.97 |
| 181 days - 2 years | 0.90 (0.80, 1.01) | 0.07 |  |  | **0.89 (0.79, 0.99)** | **0.04** |
| >2 years | **0.78 (0.64, 0.96)** | **0.02** |  |  | **0.79 (0.65, 0.97)** | **0.02** |

Model 1 = Allopurinol use + age + race + gender +Diabetes + hypertension+ Hyperlipidemia + tobacco disorder + beta blockers + diuretics + ACE inhibitors

Model 2 = Allopurinol use duration + age + race+ gender + Diabetes +hypertension+ Hyperlipidemia + tobacco disorder + beta blockers + diuretics + ACE inhibitors

CVD, Cerebrovascular disease; PVD, Peripheral vascular disease
