# Supplementary material for: Allopurinol and the risk of stroke in older adults receiving medicare
Source: BMC Neurol. 2016 Sep 7;16(1):164. doi: 10.1186/s12883-016-0692-2 (PMC5015204; doi:10.1186/s12883-016-0692-2)
Supplement: Additional file 3: Table S3. — Subgroup analyses by the type of stroke. (DOCX 89 kb) [file 12883_2016_692_MOESM3_ESM.docx]

**Additional File 3.** Subgroup analyses by the type of stroke

|  | Univariate |  | Multivariable-adjusted (model1) |  | Multivariable-adjusted (model2) |  |
| --- | --- | --- | --- | --- | --- | --- |
|  | HR (95% CI) | P-value | HR (95% CI) | P-value | HR (95% CI) | P-value |
| Hemorrhagic stroke | | | | | | |
| **Allopurinol use (Ref,** none**)** | 1.02 (0.80, 1.30) | 0.84 | 1.01 (0.79, 1.29) | 0.94 | - | - |
| **Allopurinol use duration** |  |  |  |  |  |  |
| 0 day | Ref |  |  |  | Ref |  |
| 1 - 180 days | 0.93 (0.66, 1.32) | 0.69 |  |  | 0.92 (0.65, 1.32) | 0.66 |
| 181 days – 2 years | 1.09 (0.80, 1.47) | 0.59 |  |  | 1.06 (0.78, 1.43) | 0.73 |
| >2 years | 1.04 (0.66, 1.64) | 0.87 |  |  | 1.04 (0.66, 1.64) | 0.86 |
| Ischemic stroke | | | | | | |
| **Allopurinol use (Ref,** none**)** | **0.91 (0.83, 0.99)** | **0.04** | **0.89 (0.81, 0.98)** | **0.02** | - | - |
| **Allopurinol use duration** |  |  |  |  |  |  |
| 0 day | Ref |  |  |  | Ref |  |
| 1 - 180 days | 1.01 (0.89, 1.16) | 0.84 |  |  | 1.00 (0.88, 1.14) | 0.99 |
| 181 days – 2 years | **0.88 (0.78, 0.99)** | **0.03** |  |  | **0.86 (0.76, 0.97)** | **0.01** |
| >2 years | **0.76 (0.62, 0.94)** | **0.01** |  |  | **0.77 (0.62, 0.95)** | **0.01** |

Respective ICD-9-CM codes were as follows: Hemorrhagic stroke, 430.xx or 431.xx; Ischemic stroke, 433.1x, 436.xx or 434.xx except 434.x0
